# Autoimmune Brainstem Encephalitis: An Illustrative Case and a Review of the Literature

**DOI:** 10.3390/jcm10132970

**Published:** 2021-07-01

**Authors:** Romy Zoghaib, Ali Sreij, Nancy Maalouf, Joumana Freiha, Raghid Kikano, Naji Riachi, Moussa A. Chalah, Samar S. Ayache, Rechdi Ahdab

**Affiliations:** 1 Gilbert and Rose Mary Chagoury School of Medicine, Lebanese American University, Byblos 4504, Lebanon; Romy.zoghaib@lau.edu (R.Z.); Ali.Sreij@lau.edu (A.S.); Nancy.maalouf@lau.edu.lb (N.M.); joumana.freiha@lau.edu (J.F.); Raghid.kikano@lau.edu.lb (R.K.); Naji.riachi@lau.edu.lb (N.R.); 2 Neurology Department, Lebanese American University Medical Center, Rizk Hospital, Beirut 113288, Lebanon; 3 Radiology Department, Lebanese American University Medical Center, Rizk Hospital, Beirut 113288, Lebanon; 4 Service de Physiologie-Explorations Fonctionnelles, Hôpital Henri Mondor, Assistance Publique–Hôpitaux de Paris, 51 Avenue de Lattre de Tassigny, 94010 Créteil, France; moussachalah@gmail.com (M.A.C.); samarayache@gmail.com (S.S.A.); 5 EA 4391, Excitabilité Nerveuse et Thérapeutique, Université Paris-Est Créteil, 94010 Créteil, France; 6 Hamidy Medical Center, Tripoli 1300, Lebanon

**Keywords:** brainstem encephalitis, multiple sclerosis, neuromyelitis optica spectrum disorder, autoimmune glial fibrillary acidic protein astrocytopathy, acute disseminated encephalomyelitis, Bickerstaff brainstem encephalitis, CLIPPERS, connective tissue disease, paraneoplastic syndromes

## Abstract

Autoimmune brainstem encephalitis (BSE) is a rare neurological condition with a wide range of underlying etiologies. It can be subdivided into two broad groups: a primary inflammatory disease of the central nervous system (CNS) or a brainstem disorder secondary to systemic diseases where the CNS is only one of many affected organs. Symptoms range from mild to life-threatening manifestations. Most cases respond well to immunotherapy. Therefore, broad and in-depth knowledge of the various inflammatory disorders that target the brainstem is essential for guiding the diagnostic approach and assisting in early initiation of appropriate therapy. We herein report on a case of BSE and provide an overview of the various causes of autoimmune BSE with an emphasis on the clinical manifestations and diagnostic approach.

## 1. Introduction

Brainstem (BS) lesions have been linked to an extensive variety of pathologies such as infections, tumors, and autoimmune disorders [1]. Challenges in the management of patients with BS lesions include identifying the underlying etiology, timely initiation of therapy, and defining prognosis. Inflammatory BS lesions can be classified into two main categories, either primary inflammatory diseases of the central nervous system (CNS)—in the setting of what is known as autoimmune brainstem encephalitis (BSE)—or CNS affection secondary to systemic diseases, where neurological symptoms are usually associated with other manifestations of the disease. In the latter case, CNS involvement usually occurs in the setting of an established systemic disease, and the diagnosis is usually clear-cut. By contrast, in some cases, these manifestations may precede other known symptoms of the disease rendering the diagnosis more challenging. 

Autoimmune BSE is one of the most common causes of BS dysfunction [1]. It often responds well to immunotherapy; this emphasizes the importance of an early diagnosis and treatment. With the discovery of new antibodies, novel entities causing BS lesions have been recently added to the already broad differential diagnoses. We herein report a case of BSE and provide an overview of the various causes of autoimmune BSE with an emphasis on the clinical manifestations and diagnostic approach.

## 2. Illustrative Case 

A previously healthy right-handed 41-year-old lady came to our institution with complaints of gait instability and dysarthria. Symptoms started abruptly and worsened progressively over the week before presentation. This was associated with transient horizontal diplopia that had resolved by presentation. She denied any fall, bowel or urinary disturbances, recent infection, or fever. No significant family history was reported. 

On bedside examination, the patient was lethargic and had severe dysarthria; however, speech production and comprehension were intact. Cranial nerve (CN) examination was unrevealing. Motor power was preserved in all tested muscle groups. Sensory exam in all modalities (vibration, pinprick, light touch, and temperature) was normal. On cerebellar exam, significant bilateral dysmetria was noted on finger-to-nose testing. Gait was severely ataxic, and the patient was unable to ambulate without assistance. 

**Figure 1:**
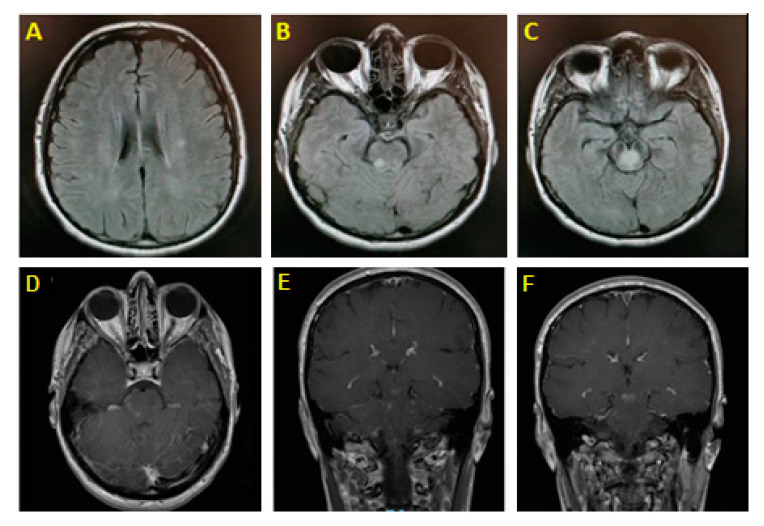
(**A**): Axial T2/FLAIR shows bilateral foci signal hyperintensities in the subcortical white matter. (**B**): Right pontine T2/FLAIR hyperintensity. (**C**): Midbrain hyperintense signal. (**D**–**F**): Subcortical lesions showing postcontrast enhancement.

Brain magnetic resonance imaging (MRI) with gadolinium showed an enhancing midbrain lesion and multiple high T2/FLAIR punctate nonenhancing subcortical white matter (WM) lesions (Figure 1). Magnetic resonance angiography of the head and neck was unrevealing. Basic metabolic panel and cerebrospinal fluid (CSF) studies (including testing for IgG index and oligoclonal bands) were unremarkable. Whole-body CT-scan was unremarkable. Visual evoked potential showed no evidence of optic neuritis. Serum anti-aquaporin-4 antibodies were negative; however, antimyelin oligodendrocyte glycoprotein (anti-MOG) antibodies returned positive (1:40). 

The diagnosis of MOG-antibodies-associated disease was set, and the patient received 1 g of intravenous (IV) Methylprednisolone daily over 5 days. At day 3 of treatment, she showed remarkable improvement in symptoms. She was discharged on 1 mg/kg of oral prednisone for 2 weeks followed by a slow taper. At follow-up 1 month later, her symptoms had entirely resolved. She was started on oral azathioprine. One year after the initial symptoms, she remains free of symptoms. 

During the last follow-up, the patient gave her written consent for the publication of this case illustration.

## 3. Etiology of Autoimmune Brainstem Encephalitis

### 3.1. Multiple Sclerosis 

Multiple sclerosis (MS) is a chronic demyelinating disease of the CNS characterized by focal inflammatory invasion causing myelin damage and secondary axonal loss [2]. BS involvement is frequent in MS and can be the inaugural symptom in 20% of cases [3]. It often presents nonspecific symptoms such as diplopia, gait disturbance, and facial sensory involvement [4]. Bilateral internuclear ophthalmoplegia (INO), facial myokymias, Uhthoff’s phenomenon, and bilateral trigeminal neuralgia with sensory deficit occurring in a young patient are also highly suggestive of MS [5]. Another rare but particular MS symptom is paroxysmal dysarthria which points toward a lower medullary pathology [3,5]. 

When a BS syndrome occurs in a patient with established MS, the diagnosis is usually that of disease relapse. Although this is the case in the majority of patients, it is crucial to keep in mind that patients on disease-modifying treatments are at higher risk of infectious complications, including infectious BSE. Progressive multifocal leukoencephalopathy (PML), a disease caused by the John Cunningham virus (JCV), should be suspected in patients with subacute and progressively worsening symptoms in the setting of immunosuppressive medications known to increase the risk of PML, such as natalizumab. BS involvement in this scenario poses a diagnostic challenge in MS patients as it may initially masquerade as an MS relapse. Early T1 hypointensity and diffusion restriction on diffusion-weighted images are highly suggestive of the diagnosis of PML; however, close MRI follow-up is highly recommended to distinguish it from new MS lesions [6]. Furthermore, an early punctate pattern of involvement in the subcortical white matter can sometimes be seen in patients with PML and may act as a useful aid for differentiating early PML from MS [7]. Listeria monocytogenes (LM) has a particular tropism for the BS and should be suspected in patients with associated headaches and fever. LM infections have been described in MS patients on dimethyl fumarate and fingolimod [8]. Early empiric treatment with ampicillin is recommended is suspected cases. 

When BSE is the first manifestation of MS, further testing is often needed, such as brain MRI, to search for evidence of dissemination in time and dissemination in space. Lesions in the BS have the propensity to affect the pons and are typically well demarcated [9]. CSF studies demonstrate intrathecal IgG synthesis and CSF-specific oligoclonal bands in most patients. CSF analysis is also helpful in excluding alternative etiologies. When no supportive findings can be found, a yearly follow-up study is recommended. Around 53–60% of patients with BS syndromes consequently develop MS [10]. Early diagnosis and prompt treatment improve the long-term prognosis of MS which is why accurate and timely detection is crucial. 

Tumefactive demyelinating lesions are a known manifestation of MS. These are rarely found in the BS and pose a diagnostic challenge if no other clinical or radiological signs are found [11]. BS biopsy may be the only reliable way to exclude a BS tumor. 

MS relapses are treated with a short course of high-dose IV methylprednisolone. Long-term management encompasses a wide selection of disease-modifying therapies that effectively suppress or modulate the immune function [12]. These include immunomodulators such as interferons, fumarates, glatiramer acetate, teriflunomide, fingolimod, newer generation siponimod and ozanimod, and monoclonal antibodies such as natalizumab, ocrelizumab, alemtuzumab, and ofatumumab [13]. The choice of treatment depends on multiple factors, notably the disease course, disease activity, lesion load, and patient preference. Mesenchymal stem cells and autologous bone marrow transplants have also been studied for the treatment of MS [13].

### 3.2. Neuromyelitis Optica Spectrum Disorder and MOG-Antibodies-Associated Disease

NMOSD is an inflammatory demyelinating disease of the CNS with a predilection for areas rich in aquaporin-4 (AQP4) membrane channel. These include the optic nerves, spinal cord, diencephalon, periventricular structures, and parts of the BS [14]. In total, 73–90% of patients with NMOSD have serum AQP4 antibodies (i.e., AQP4+Ab NMOSD), while 42% of seronegative patients (i.e., AQP4-Ab NMOSD) have anti-MOG antibodies [15]. Anti-MOG-associated demyelinating disease primarily affects the myelin sheath and oligodendrocytes [16]. The clinical spectrum ranges from acute demyelinating encephalomyelitis (more common in children) to recurrent optic neuritis, myelitis, encephalomyelitis, and BS syndromes in adults [17]. 

BS involvement occurs in one third of patients with AQP4+Ab NMOSD. As with NMOSD prevalence, which seems to vary according to ethnicity [18], brain or BS involvement at disease onset seems to be more frequent in Afro-American/Afro-European patients, followed by Asian patients and to a lesser extent by Caucasian patients, according to one study [19]. The most characteristic presentation is uncontrollable vomiting and hiccups often misdiagnosed as gastrointestinal (GI) upset [20]. These can be the first manifestation of the disease in up to 12% of patients [20,21]. GI symptoms are usually associated with lesions in the area postrema, an AQP4 rich emetic reflex center in the medulla [22]. Lesions in this location were found to lack demyelination and necrosis commonly seen in afflicted optic nerves and spinal cord, a feature that makes these areas more prone to full recovery when treated promptly [23]. Other signs localizing to the BS are seen in 41.3% of patients and include diplopia/ocular movement disorders, facial dysesthesia, and trigeminal neuralgia, dysgeusia, facial paralysis, hearing loss, tinnitus, vertigo, and dysarthria/dysphagia [14,20]. Neuropathic pruritus, defined as an itch occurring in the absence of a pruritogenic substance, is another possible manifestation of BS involvement and may be the presenting symptom of NMOSD [24]. This phenomenon could be attributed to a demyelinating lesion involving the dorsal root ganglion of the spinal cord or the spinal nucleus of the trigeminal nerve in the BS [25]. Patients with medullary involvement may also present with life-threatening respiratory failure [14]. On MRI, BS lesions often involve the dorsal medulla, and they are typically asymmetrical with ill-defined margins [9]. In anti-MOG-associated diseases, BS involvement occurs in about 7% and predominantly affects the pons, ranging from mild symptoms such as cranial neuropathies, intractable vomiting, internuclear ophthalmoplegia, and limb and gait ataxia to hypoventilation and respiratory compromise [26,27]. Subclinical MRI lesions in the BS have been detected in a subset of patients [27]. 

The clinical manifestations of NMOSD overlap with those of MS. As such, testing for serum AQP4 antibodies should be considered in the following settings: (a) patients presenting with longitudinally extensive transverse myelitis (involving more than three vertebral levels); (b) patients with atypical optic neuritis (i.e., bilateral optic nerve or chiasmal involvement, long lesion of the optic nerve, poor recovery, or inadequate response to steroids); (c) patients with diencephalic syndrome and nonspecific MRI findings; (d) patients with unexplained encephalopathy; and (e) patients with area postrema syndrome (APS) [14]. CSF analysis usually shows mild to moderate pleocytosis with high protein content. Oligoclonal bands in CSF are only found in less than 20% of AQP4+NMOSD and anti-MOG patients [28]. Involvement of the anterior rather than the posterior portion of optic nerves with sparing of the optic tract and chiasm, myelitis with predominant involvement of the ventral grey matter without contrast enhancement, and/or “fluffy” lesions in the white and grey matter should prompt testing for anti-MOG titers, specifically in the context of AQP4 antibodies seronegativity [17]. 

First-line treatment of acute relapses of NMOSD includes IV methylprednisolone followed by oral steroids [29]. If the latter proves unsuccessful, plasmapheresis and human immunoglobulin may be considered [29]. Since NMOSD is a highly active inflammatory disease, prevention of relapses should be undertaken with chronic use of immunosuppressants such as azathioprine, mycophenolate mofetil, methotrexate, mitoxantrone, cyclophosphamide, and monoclonal antibodies (e.g., inebilizumab, eculizumab, rituximab, tocilizumab, and aquaporumab) [30]. Disease-modifying drugs used in MS are ineffective in the treatment of NMOSD and may be detrimental, which is why an accurate diagnosis is crucial [28]. Treatment of anti-MOG demyelinating disease consists of a similar approach with steroids or plasma exchange in the acute setting and disease-modifying therapy for the management of relapsing disease [17]. BS involvement in anti-MOG disease usually indicates a more aggressive course and warrants prophylactic long-term treatment [27]. As opposed to AQP4+Ab NMOSD, for most patients with anti-MOG antibodies, the risk of future disability is low, owing to good initial recovery from clinical attacks [16].

### 3.3. Autoimmune Glial Fibrillary Acidic Protein Astrocytopathy 

Autoimmune glial fibrillary acidic protein astrocytopathy (GFAP) is a recently described autoimmune astrocytopathy affecting the CNS characterized by relapsing meningoencephalomyelitis with GFAP-IgG in serum or CSF [31]. Clinical features of the disease include encephalopathy, drug-resistant seizures, meningitis-like signs, and psychiatric symptoms. Initial flu-like symptoms are seen in 40–60% of cases. GFAP can occur in the context of paraneoplastic diseases, and a search for an underlying malignancy is warranted [32]. Isolated BSE is not a classical manifestation of the illness, although BS lesions have been described on MRI. An overlap syndrome with both MOG-IgG and GFAP-IgG present in serum and CSF has been reported in a young patient presenting with BSE [31]. Another case of a young patient presenting longitudinally extensive transverse myelitis with medulla oblongata involvement in the presence of both GFAP-IgG and AQP4 antibodies has also been described [33]. Optic neuritis and myelitis can also occur as part of the spectrum and can be differentiated from similar presentations of NMOSD, in that the myelitis manifests mainly with sensory and mild motor signs and optic neuritis with blurred vision secondary to optic disc edema [32]. Brain MRI typically shows a pattern of radial linear periventricular gadolinium-enhancement [31]. This entity usually responds very well to steroids in the acute setting but often requires long-term treatment with steroid-sparing drugs, such as mycophenolate mofetil, azathioprine, rituximab, and cyclophosphamide [32], given its relapsing nature.

### 3.4. Acute Disseminated Encephalomyelitis/Acute Hemorrhagic Leukoencephalitis

Acute disseminated encephalomyelitis (ADEM) is a monophasic demyelinating disorder of the CNS, often precipitated by an infectious event or vaccination. ADEM has been primarily described in pediatric populations; however, it has been reported in adults [34]. It is often characterized by an acute clinical onset accompanied by fever and meningismus and primarily implicates the brain white matter and spinal cord [35]. ADEM frequently involves the infratentorial compartment particularly in children presenting with ataxia, oculomotor disturbances, and dysarthria [28]. Serum testing is positive for anti-MOG antibodies in 40% of children and is often linked to a better clinical and radiological outcome [36]. MRI lesions are usually asymmetric with ill-defined margins and minimal mass effect and characteristically involve the BS, particularly the ventral midbrain [9]. Their location correlates with clinical symptoms and signs [37]. Other involved areas include the basal ganglia, thalami, and cortex [34]. ADEM presenting as isolated BS lesions, although a rare occurrence, has been described in pediatric patients [38]. 

A rare variant of ADEM known as acute hemorrhagic leukoencephalitis (AHLE) is more commonly seen in adults. This variant is a highly aggressive disease and often results in death early after onset. AHLE can rarely present as isolated BS involvement. Atherton and colleagues described a case of AHLE presenting with acute rhombencephalitis with MRI evidence of hemorrhagic foci in the BS following infection with a coxsackievirus strain [39]. This case highlights the importance of considering this entity in the differential diagnosis of acute progressive BS syndrome. 

Treatment of ADEM and AHLE consists of eradicating the inciting pathogen when applicable, followed by a course of steroids [39]. Alternatively, immunoglobulins, plasmapheresis, and cytostatic drugs could be considered in patients who do not respond to steroid therapy [34]. Prognosis is usually favorable in ADEM as opposed to its more fulminant AHLE variant. 

### 3.5. Bickerstaff Brainstem Encephalitis 

Bickerstaff brainstem encephalitis (BBE) is a condition characterized by a triad of ataxia, impaired consciousness/ hypersomnolence, and symmetrical ophthalmoplegia associated with the production of anti-GQ1b antibodies [40,41]. The diagnosis of a probable BBE requires the presence of a subacute onset (less than 4 weeks) of all three of the abovementioned clinical symptoms after the exclusion of alternative etiologies, while a definite BBE diagnosis is made in the context of positive anti-GQ1b antibodies [42]. Although the presence of ophthalmoplegia and ataxia is mandatory for the definite diagnosis of BBE, it has been suggested that BBE may sometimes present as “incomplete” in the context of an anti-GQ1b positive disease, and its frequency may be underestimated [43,44]. Additional symptoms and signs include pupillary abnormalities, facial weakness or dysesthesias, and bulbar palsy in up to 50% of patients [45]. Other rare manifestations include pseudobulbar affect, optic neuropathy, APS, and decorticate-like posturing [41,46,47]. The disease follows a monophasic course with subacute onset and a favorable outcome in most cases [40].

The disease pathophysiology is far from being fully understood. A postinfectious process has been proposed given the frequent presence of an antecedent infection. Laboratory studies often show evidence of albumin-cytologic dissociation in CSF [40]. The presence of anti-GQ1b IgG antibodies is specific for this disorder but can be absent in 30% of cases [40,41]. These antibodies can also be seen in an ataxic variant of Guillain–Barré syndrome, known as Miller–Fisher syndrome, characterized by ophthalmoplegia and areflexia. Imaging is usually unremarkable, but T2-weighted BS abnormalities can sometimes be seen [48]. 

Patients with BBE are often treated with a course of IV immunoglobulins (IVIG) as monotherapy or in combination with steroids [49]. Plasmapheresis has also been successfully used in this setting. Generally, the disease entails a good prognosis with early treatment, and the majority of patients go on to have a complete recovery [40].

### 3.6. CLIPPERS

CLIPPERS (i.e., chronic lymphocytic inflammation with pontine perivascular enhancement responsive to steroids), as the term implies, is a rare chronic inflammatory disease of the CNS characterized by distinct BS involvement with prominent clinical and radiological response to steroids [50]. The disease typically manifests as a subacute onset cerebellar and BS syndrome with ataxia, dysarthria, diplopia, and/or facial sensory disturbances [51]. Other nonspecific BS signs have also been described less frequently, such as dysgeusia, oculomotor abnormalities (i.e., oculomotor palsies, gaze palsy, internuclear ophthalmoplegia, one-and-a-half syndrome, disturbances of saccadic eye, and slow eye pursuit), nystagmus, cranial neuropathies (i.e., CN VII, VIII, IX, XII), hiccups, and nausea [50,51]. Additional features include pseudobulbar affect and spinal cord syndrome (i.e., pyramidal signs, paraparesis, spasticity, and sphincter dysfunction) with or without cognitive deficits [52].

In the absence of a specific serum or CSF biomarker, the diagnosis of CLIPPERS is based on radiological features and exclusion of alternative etiologies. MRI shows a distinctive punctate curvilinear pattern of patchy gadolinium enhancement ‘peppering’ the pons, BS, cerebellum, and spinal cord [51]. Although highly suggestive of the disease in the proper clinical context, similar radiological features can be observed in other inflammatory disorders such as primary angiitis of the CNS, primary CNS lymphoma, and CNS lymphomatoid granulomatosis [53]. The findings of homogenous enhancement of <3 mm lesions in the pons and cerebellum showing no significant mass effect or vasogenic edema can help distinguish CLIPPERS from mimickers [54]. CSF analysis is usually nonrevealing with occasional evidence of a mild pleocytosis and protein elevation. Oligoclonal bands have been detected in some patients [52]. 

A classical feature of CLIPPERS is its sensitivity to steroid therapy and its remarkable clinical and radiological improvement within a few days of initiation of treatment. In fact, the failure of steroids to produce a rapid resolution of radiological findings warrants consideration of an alternative diagnosis [52]. Management of an acute attack should be initiated as soon as possible after exclusion of alternative diagnosis and begins with a short course of IV methylprednisolone followed by tapering doses of oral Prednisone [55]. It is important to note that early taper of steroids was found to be associated with recurrence of symptoms and radiological progression [50,51]. In the absence of steroids, the disease follows a relapsing course with a mean annualized relapse rate of 0.5. Patients were maintained on chronic steroid therapy above 20 mg often remain relapse-free. Data regarding the efficacy of steroid-sparing agents are scarce and based on case reports and case series. Agents with possible efficacy include methotrexate, cyclophosphamide, hydroxychloroquine, azathioprine, and tocilizumab [55,56]. The duration of maintenance treatment is still not yet clearly established and should be tailored to each case. Furthermore, follow-up MRI studies are recommended to rule out potential malignant evolution, since cases of early-stage CNS lymphoma presenting as CLIPPERS have been described [57].

### 3.7. Connective Tissue Disease and Vasculitis 

BSE can occur in the setting of various systemic autoimmune disorders. Establishing the diagnosis of BSE is relatively straightforward when the primary illness is already labeled; however, this becomes significantly more challenging when BSE presents as the first and only manifestation of the systemic disorder. Failure to consider connective tissue diseases (CTD) and vasculitis as an underlying etiology can significantly delay treatment and lessen the probability of full recovery. 

Behçet disease (BD) is characterized by a diagnostic triad of recurrent oral aphthous ulcers, genital ulcers, and uveitis [54]; however, this triad may sometimes be absent at initial presentation. Neurological complications, categorized as having either parenchymal or secondary nonparenchymal involvement, occur in roughly 10% of patients. These may precede the other manifestations of the disease in 6% of cases [58]. BD has a tropism for the BS, and lesions tend to localize to the posterior aspect of the midbrain–diencephalic junction, while characteristically sparing the red nucleus [55,56]. The clinical manifestations usually consist of motor and sensory symptoms with minimal ataxia [59]. CSF analysis often displays mildly elevated protein levels and the absence of oligoclonal bands. Occasionally, pleocytosis with white cell count exceeding 100 cells/μl can also be seen [58]. In addition, 30–50% of patients have a relapsing course despite initial response to steroids [60]. To note, other entities such as anti-MOG encephalitis may sometimes mimic presentations of neuro-Behçet disease [61]. Other types of systemic vasculitis can also involve the CNS such as eosinophilic granulomatosis with polyangiitis (Churg–Strauss syndrome), polyarteritis nodosa, and granulomatosis with polyangiitis (Wegener’s granulomatosis). As opposed to BD, these disorders rarely involve the BS.

CNS disorders can be seen in the context of different CTD, with some causing symptoms of BS dysfunction [62]. Sjögren’s syndrome (SS) causes a broad spectrum of neurological manifestations, including BSE or focal BS lesions, which may present variable symptoms such as intractable vomiting, dysphagia, and slurred speech [63]. In some cases, these symptoms may precede the diagnosis of SS by up to 2 years [63,64]. SS may also mimic MS in its relapsing–remitting clinical course as well as its imaging features, posing a further challenge in diagnosis [65]. Moreover, systemic lupus erythematosus (SLE.) also commonly manifests with a wide range of neurological disorders. As with other autoimmune systemic diseases, it may rarely cause BS dysfunction occurring in isolation and preceding other manifestations of the disease [66,67]. It is also important to note that SLE and SS can coexist with NMOSD. This scenario should be considered when myelitis and/or optic neuritis occur in the setting of a known systemic inflammatory disease [68]. The coexistence of SS and NMOSD can be attributed to the presence of an autoimmune predisposition rather than a complication of the systemic disease and does not necessarily imply a poorer outcome [69]. Patients with SS-NMOSD are often treated with steroids alone or in combination with azathioprine [69]. Tocilizumab has also been used as a potential treatment for patients with SS and NMOSD [70].

### 3.8. Paraneoplastic Syndromes 

Paraneoplastic diseases of the CNS are a group of immune-mediated disorders with a wide range of clinical manifestations. Although BSE is not commonly implicated in paraneoplastic syndromes, some antibodies have been linked to BS dysfunction such as anti-Hu, anti-Ma2, anti-Ri, Kelch-like protein-11 (KLHL11) IgG, and Leucine Zipper 4 (LUZP4) IgG antibodies [71,72,73]. These entities have been classified as intermediate-risk phenotypes as per the diagnostic criteria for paraneoplastic neurologic syndromes [74]. The onset is typically subacute, with rapid worsening and often devastating consequences.

Anti-Hu antibodies are classically associated with paraneoplastic encephalomyelitis, almost always in the setting of small cell lung carcinoma. Early in the disease course, more focal syndromes can be seen, such as limbic encephalitis, cerebellar degeneration, or BSE [75,76]. BSE is the predominant syndrome in 11% of cases [77]. This entity has a distinct tropism for the medulla and patients often present with dysphagia, dysarthria, and hypoventilation [76]. Other manifestations may include CN VI/VII palsy, vertical nystagmus, and ataxia [76]. MRI and CSF analysis are typically normal. The prognosis is usually poor despite immunotherapy and treatment of the underlying tumor. 

Anti-Ma2 is typically a disease of young men with testicular germ-cell tumors, while in the older population, it is more commonly associated with lung and breast malignancies [77,78]. Anti-Ma2-associated encephalitis characteristically affects the limbic system, hypothalamus, and BS [75]. Midbrain involvement commonly manifests as supranuclear vertical gaze palsy and oculomotor nuclei involvement. Other characteristic symptoms include excessive daytime sleepiness, narcolepsy, cataplexy, rapid eye movement (REM)-sleep abnormalities, hyperphagia, and memory impairment. Brain MRI may show T2-hyperintense lesions in the superior colliculi and periaqueductal region. Roughly one third of patients can be expected to respond to tumor resection and immunotherapy [78].

Anti-Ri antibodies also have a tropism for the BS [79]. These are the least common paraneoplastic autoantibodies, primarily encountered in patients with breast and ovarian cancers. Patients typically present with signs of BS, cerebellar, and spinal cord dysfunction. BS dysfunction often manifests as an opsoclonus-myoclonus syndrome, ophthalmoplegia, and facial sensory symptoms [79,80]. Treatment of underlying cancer can also lead to a decrease in the antibody titer and improvement of symptoms [79]. 

Newly described paraneoplastic antibodies (i.e., KLHL11 IgG and LUZP4 IgG) have also been implicated in patients with BS dysfunction [71,72]. KLHL11 antibodies are generally associated with testicular germ cell tumors in men and present the clinical picture of a rhombencephalitis typically with hearing loss and tinnitus [71]. Brain MRI, although normal early in the disease, may demonstrate T2/FLAIR abnormalities in the BS or limbic system [71]. Encephalitis associated with KLHL11 IgG is generally refractory to treatment, and only 25% of patients respond to immunotherapy [71]. LUZP4 antibodies, on the other hand, typically are present in patients with germ cell tumors (commonly seminomas) and manifest with polyradicular and anterior horn cell involvement, as well as rhombencephalitis [72]. Coexistence of LUZP4 and KLHL11 antibodies has been described and usually entails a poorer neurological outcome [72].

The different etiologies of autoimmune BSE along with their clinical characteristics, neuroimaging findings, antibodies profiles, and treatments are summarized in Table A1.

## 4. Conclusions

Autoimmune BSE is only one of many causes of BS dysfunction. For instance, various disease states may also target the BS and should be included in the differential diagnoses of BSE. These include but are not limited to osmotic demyelination syndrome commonly in the context of hyponatremia [81], hypertensive encephalopathy in patients presenting with severe hypertension, visual symptoms and seizures [82], and Wernicke encephalopathy in patients with thiamine deficiency and opthalmoparesis [83]. Furthermore, the presence of fever in an immunocompromised state should always raise the suspicion of an underlying infectious cause. While infectious and autoimmune etiologies are more likely seen in young patients, neoplastic diseases are more prevalent in older populations [59]. Given this wide array of causes of BS dysfunction, awareness of the multiple disorders that may manifest with BS lesions and a comprehensive approach to diagnosis are essential for proper management. The importance of early recognition of autoimmune BSE cannot be overemphasized since the damage is often reversible with appropriate therapy. Long-term management is dependent on the precise underlying etiology. Although some entities follow a monophasic course, others are prone to relapses. Early institution of immunotherapy after the acute stage can have a significant impact on the likelihood of recurrent inflammatory events and the long-term prognosis. 

## Data Availability

All data analyzed during this work are included in this article.

## Appendix A

**Table A1:** Summary of Inflammatory Brainstem Diseases with Clinical Features, Imaging, Antibodies, and Treatments.

Disease		Characteristic Features	Brain MRI Findings	Antibodies	Treatment
**MS** **[9,13]**		Nonspecific symptoms	Periventricular white matter lesions, Dawson fingers, DIT + DIS, pontine predilection	-	DMDs
**NMOSD** **[14,15,22,30]**		Intractable vomiting, hiccups, optic neuritis, myelitis	LETM, optic nerve lesions, BS lesions	AQP4-Ab	1st line: steroids 2nd line: Azathioprine, Mycophenolate mofetil, Methotrexate, Mitoxantrone, Cyclophosphamide, and monoclonal antibodies
**Anti-MOG** **[17,27]**		Bilateral optic neuritis, LETM, BS encephalitis	Poorly demarcated, ‘fluffy’ lesions of grey and white matter Non-enhancing hyperintensities in ventral grey matter of spinal cord Anterior optic nerve involvement sparing the chiasm	MOG-IgG	Acute attack: steroids/PLEX Relapse: DMDs, immunosuppressants
**GFAP** **Astrocytopathy** **[31]**		Drug-resistant seizures, meningoencephalomyelitis, psychiatric symptoms	Radial linear periventricular gadolinium enhancement	GFAP-IgG	Acute: steroids Long-term: mycophenolate, azathioprine, rituximab, cyclophosphamide
**ADEM/AHLE** **[9,34,35,36]**		Post-viral presentation, fever, meningismus	Asymmetric ill-defined lesions, ventral midbrain	MOG-IgG	Eradicate pathogen Steroids, IVIg, PLEX
**Bickerstaff BS Encephalitis** **[16,40,48]**		Ataxia, impaired consciousness and ophthalmoplegia	Unremarkable, occasional T2 BS abnormalities	Anti-GQ1b ganglioside antibodies	IVIg, steroids, PLEX
**CLIPPERS** **[50,51,55]**		Drastic clinical and radiological response to steroid	Punctate patchy enhancing pontine lesions	-	Steroids Steroid-sparing as add on
**Behcet’s Disease** **[60]**		Oro-genital ulcers, uveitis, skin lesions	Lesions in posterior midbrain-diencephalic junction, sparing the red nucleus	-	Steroids Immunosuppressants
**Sjögren Syndrome** **[65]**		PNS involvement, Sicca Symptoms, MS-like presentation after age 50	Nonspecific, MS-like lesions	Anti-SSA Anti-SSB	Steroids + immunosuppressants, Tocilizumab
**Systemic Lupus Erythematosus** [66]		Rash, Neuropsychiatric features, young women	Non-specific	ANA, Anti-DNA antibodies	Immunosuppressants
**Paraneoplastic** **Syndromes** **[71,72,73,75,79,80]**	**Anti-Hu**	Small cell lung carcinoma Medullary dysfunction	Normal	Anti-Hu antibodies	Immunotherapy and treatment of underlying tumor
	**Anti-Ma2**	Testicular germ-cell tumors Midbrain dysfunction	T2-Hyperintense lesions in superior colliculi & periaqueductal region	Anti-Ma2 antibodies	
	**Anti-Ri**	Breast/Ovarian cancers Opsoclonus-Myoclonus	Normal	Anti-Ri antibodies	
	**KLHL11**	Testicular germ cell tumor Hearing loss and tinnitus	T2/FLAIR abnormalities in BS and limbic system	KLHL11 IgG	Immunotherapy and treatment of underlying tumor
	**LUZP4**	Germ cell tumor (seminoma) Lower motor neuron involvement, encephalitis, polyradiculopathy	T2/FLAIR abnormalities	LUZP4 IgG	Immunotherapy and treatment of underlying tumor

ADEM: Acute Disseminated Encephalomyelitis; AHLE: Acute Hemorrhagic Leukoencephalitis; ANA: Anti-Nuclear Antibody; AQP4-Ab: Aquaporin-4 Antibodies; BS: Brainstem; CLIPPERS: Chronic Lymphocytic Inflammation with Pontine Perivascular Enhancement Responsive to Steroids; DIT: Dissemination in Time; DIS: Dissemination in Space; DMDs: Disease Modifying Drugs; FLAIR: Fluid Attenuated Inversion Recovery; GFAP: Glial Fibrillary Acidic Protein; IVIg: Intravenous Immunoglobulins; KLHL11: Kelch-like protein-11; LETM: Longitudinally Extensive Transverse Myelitis; LUZP4: Leucine Zipper Protein 4; MOG: Myelin oligodendrocyte glycoprotein; MS: Multiple Sclerosis; NMOSD: Neuromyelitis Optica Spectrum Disorders; PLEX: Plasma exchange; PNS: Peripheral Nervous System.

## References

[1] Tan I.L., Mowry E.M., Steele S.U., Pardo C.A., McArthur J.C., Nath A., Venkatesan A. (2013). Brainstem encephalitis: Etiologies, treatment, and predictors of outcome. J. Neurol..

[2] Compston A., Coles A. (2008). Multiple sclerosis. Lancet.

[3] Habek M. (2013). Evaluation of brainstem involvement in multiple sclerosis. Expert Rev. Neurother..

[4] Sastre-Garriga J., Tintore M., Nos C., Tur C., Rio J., Tellez N., Castillo J., Horga A., Perkal H., Comabella M. (2010). Clinical features of CIS of the brainstem/cerebellum of the kind seen in MS. J. Neurol..

[5] Freiha J., Riachi N., Chalah M.A., Zoghaib R., Ayache S.S., Ahdab R. (2020). Paroxysmal symptoms in multiple sclerosis—A review of the literature. J. Clin. Med..

[6] Tortorella C., Direnzo V., D’Onghia M., Trojano M. (2013). Brainstem PML lesion mimicking MS plaque in a 402 natalizumab-treated MS patient. Neurology.

[7] Hodel J., Darchis C., Outteryck O., Verclytte S., Deramecourt V., Lacour A., Zins M., Pruvo J.-P., Vermersch P., Leclerc X. (2016). Punctate pattern: A promising imaging marker for the diagnosis of natalizumab-associated PML. Neurology.

[8] Tecellioglu M., Kamisli O., Kamisli S., Erdogmus U.A., Özcan C. (2019). Listeria monocytogenes rhombencephalitis in a patient with multiple sclerosis during fingolimod therapy. Mult. Scler. Relat. Disord..

[9] Lu Z., Zhang B., Qiu W., Kang Z., Shen L., Long Y., Huang J., Hu X. (2011). Comparative brain stem lesions on MRI of acute disseminated encephalomyelitis, neuromyelitis optica, and multiple sclerosis. PLoS ONE.

[10] Tintore M., Rovira A., Arrambide G., Mitjana R., Río J., Auger C., Nos C., Edo M.C., Castilló J., Horga A. (2010). Brainstem lesions in clinically isolated syndromes. Neurology.

[11] Mitsutake A., Sato T., Katsumata J., Nakamoto F.K., Seki T., Maekawa R., Hideyama T., Shimizu J., Shiio Y. (2019). Tumefactive multiple sclerosis which initially presented with brainstem encephalitis with a long-term follow-up. Mult. Scler. Relat. Disord..

[12] Hauser S.L., Cree B.A.C. (2020). Treatment of multiple sclerosis: A review. Am. J. Med..

[13] Gholamzad M., Ebtekar M., Ardestani M.S., Azimi M., Mahmodi Z., Mousavi M.J., Aslani S. (2019). A comprehensive review on the treatment approaches of multiple sclerosis: Currently and in the future. Inflamm. Res..

[14] Lana-Peixoto M.A., Talim N. (2019). Neuromyelitis optica spectrum disorder and anti-MOG Syndromes. Biomedicines.

[15] Hamid S.H.M., Whittam D., Mutch K., Linaker S., Solomon T., Das K., Bhojak M., Jacob A. (2017). What proportion of AQP4-IgG-negative NMO spectrum disorder patients are MOG-IgG positive? A cross sectional study of 132 patients. J. Neurol..

[16] Reindl M., Waters P. (2019). Myelin oligodendrocyte glycoprotein antibodies in neurological disease. Nat. Rev. Neurol..

[17] Hegen H., Reindl M. (2020). Recent developments in MOG-IgG associated neurological disorders. Adv. Neurol. Disord..

[18] Hor J.Y., Asgari N., Nakashima I., Broadley S.A., Leite M.I., Kissani N., Jacob A., Marignier R., Weinshenker B.G., Paul F. (2020). Epidemiology of neuromyelitis optica spectrum disorder and its prevalence and incidence worldwide. Front. Neurol..

[19] Kim S.H., Mealy M.A., Levy M., Schmidt F., Ruprecht K., Paul F., Ringelstein M., Aktas O., Hartung H.P., Asgari N. (2018). Racial differences in neuromyelitis optica spectrum disorder. Neurology.

[20] Kremer L., Mealy M., Jacob A., Nakashima I., Cabre P., Bigi S., Paul F., Jarius S., Aktas O., Elsone L. (2014). Brainstem manifestations in neuromyelitis optica: A multicenter study of 258 patients. Mult. Scler..

[21] Apiwattanakul M., Popescu B.F., Matiello M., Weinshenker B.G., Lucchinetti C.F., Lennon V.A., McKeon A., Carpenter A.F., Miller G.M., Pittock S.J. (2010). Intractable vomiting as the initial presentation of neuromyelitis optica. Ann. Neurol..

[22] Iorio R., Lucchinetti C.F., Lennon V.A., Farrugia G., Pasricha P.J., Weinshenker B.G., Pittock S.J. (2013). Intractable nausea and vomiting from autoantibodies against a brain water channel. Clin. Gastroenterol. Hepatol..

[23] Prabhu M.M., Agrawal U. (2019). Intractable vomiting and hiccups: An atypical presentation of neuromyelitis optica. Cureus.

[24] Lee S., Lee H.-S., Baek S.-H. (2010). Paroxysmal pruritus as the first relapsing symptom of neuromyelitis optica. Neurol. Asia.

[25] Elsone L., Townsend T., Mutch K., Das K., Boggild M., Nurmikko T., Jacob A. (2013). Neuropathic pruritus (itch) in neuromyelitis optica. Mult. Scler..

[26] Cobo-Calvo A., Vukusic S., Marignier R. (2019). Clinical spectrum of central nervous system myelin oligodendrocyte glycoprotein autoimmunity in adults. Curr. Opin. Neurol..

[27] Jarius S., Kleiter I., Ruprecht K., Asgari N., Pitarokoili K., Borisow N., Hummert M.W., Trebst C., Pache F., Winkelmann A. (2016). MOG-IgG in NMO and related disorders: A multicenter study of 50 patients. Part 3: Brainstem involvement-frequency, presentation and outcome. J. Neuroinflamm..

[28] Wingerchuk D.M., Banwell B., Bennett J.L., Cabre P., Carroll W., Chitnis T., de Seze J., Fujihara K., Greenberg B., Jacob A. (2015). International consensus diagnostic criteria for neuromyelitis optica spectrum disorders. Neurology.

[29] Wu Y., Zhong L., Geng J. (2019). Neuromyelitis optica spectrum disorder: Pathogenesis, treatment, and experimental models. Mult. Scler. Relat. Disord..

[30] Akaishi T., Nakashima I. (2017). Efficiency of antibody therapy in demyelinating diseases. Int. Immunol..

[31] Ding J., Ren K., Wu J., Li H., Sun T., Yan Y., Guo J. (2020). Overlapping syndrome of MOG-IgG-associated disease and autoimmune GFAP astrocytopathy. J. Neurol..

[32] Kunchok A., Zekeridou A., McKeon A. (2019). Autoimmune glial fibrillary acidic protein astrocytopathy. Curr. Opin. Neurol..

[33] Li X.L., Han J., Zhao H.T., Long Y.M., Zhang B.W., Wang H.Y. (2020). Autoimmune glial fibrillary acidic protein astrocytopathy with lesions distributed predominantly in the entire spinal cord. Ther. Adv. Neurol. Disord..

[34] Schwarz S., Mohr A., Knauth M., Wildemann B., Storch-Hagenlocher B. (2001). Acute disseminated encephalomyelitis: A follow-up study of 40 adult patients. Neurology.

[35] Hardy T.A., Reddel S.W., Barnett M.H., Palace J., Lucchinetti C.F., Weinshenker B.G. (2016). Atypical inflammatory demyelinating syndromes of the CNS. Lancet Neurol..

[36] Baumann M., Sahin K., Lechner C., Hennes E.M., Schanda K., Mader S., Karenfort M., Selch C., Hausler M., Eisenkolbl A. (2015). Clinical and neuroradiological differences of paediatric acute disseminating encephalomyelitis with and without antibodies to the myelin oligodendrocyte glycoprotein. J. Neurol. Neurosurg. Psychiatry.

[37] Atlas S.W., Grossman R.I., Goldberg H.I., Hackney D.B., Bilaniuk L.T., Zimmerman R.A. (1986). MR diagnosis of acute disseminated encephalomyelitis. J. Comput. Assist. Tomogr..

[38] Alper G., Sreedher G., Zuccoli G. (2013). Isolated brain stem lesion in children: Is it acute disseminated encephalomyelitis or not?. AJNR Am. J. Neuroradiol..

[39] Atherton D.S., Perez S.R., Gundacker N.D., Franco R., Han X. (2016). Acute disseminated encephalomyelitis presenting as a brainstem encephalitis. Clin. Neurol. Neurosurg..

[40] Shahrizaila N., Yuki N. (2013). Bickerstaff brainstem encephalitis and Fisher syndrome: Anti-GQ1b antibody syndrome. J. Neurol. Neurosurg. Psychiatry.

[41] Horton E., Krishnamoorthy S., Reynolds L. (2014). Bickerstaff’s encephalitis. BMJ Case Rep..

[42] Graus F., Titulaer M.J., Balu R., Benseler S., Bien C.G., Cellucci T., Cortese I., Dale C.R., Gelfand M.J., Geschwind M. (2016). A clinical approach to diagnosis of autoimmune encephalitis. Lancet Neurol..

[43] Kuwabara S., Misawa S., Mori M. (2013). Bickerstaff brainstem encephalitis: More common than we think?. J. Neurol. Neurosurg. Psychiatry.

[44] Wakerley B.R., Soon D., Chan Y.C., Yuki N. (2013). Atypical Bickerstaff brainstem encephalitis: Ataxic hypersomnolence without ophthalmoplegia. J. Neurol. Neurosurg. Psychiatry.

[45] Ito M., Kuwabara S., Odaka M., Misawa S., Koga M., Hirata K., Yuki N. (2008). Bickerstaff′s brainstem encephalitis and Fisher syndrome form a continuous spectrum: Clinical analysis of 581 cases. J. Neurol..

[46] Kurihara M., Bannai T., Otsuka J., Kawabe Matsukawa M., Terao Y., Shimizu J., Tsuji S. (2018). Optic neuropathy and decorticate-like posture as presenting symptoms of Bickerstaff′s brainstem encephalitis: A case report and literature review. Clin. Neurol. Neurosurg..

[47] Zeiner P.S., Brandhofe A., Müller-Eschner M., Steinmetz H., Pfeilschifter W. (2018). Area postrema syndrome as frequent feature of Bickerstaff brainstem encephalitis. Ann. Clin. Transl. Neurol..

[48] Koga M., Kusunoki S., Kaida K., Uehara R., Nakamura Y., Kohriyama T., Kanda T. (2012). Nationwide survey of patients in Japan with Bickerstaff brainstem encephalitis: Epidemiological and clinical characteristics. J. Neurol. Neurosurg. Psychiatry.

[49] Yoshikawa K., Kuwahara M., Morikawa M., Kusunoki S. (2020). Bickerstaff brainstem encephalitis with or without anti-GQ1b antibody. Neurol. Neuroimmunol. Neuroinflamm..

[50] Dudesek A., Rimmele F., Tesar S., Kolbaske S., Rommer P.S., Benecke R., Zettl U.K. (2014). CLIPPERS: Chronic lymphocytic inflammation with pontine perivascular enhancement responsive to steroids. Review of an increasingly recognized entity within the spectrum of inflammatory central nervous system disorders. Clin. Exp. Immunol..

[51] Pittock S.J., Debruyne J., Krecke K.N., Giannini C., van den Ameele J., De Herdt V., McKeon A., Fealey R.D., Weinshenker B.G., Aksamit A.J. (2010). Chronic lymphocytic inflammation with pontine perivascular enhancement responsive to steroids (CLIPPERS). Brain.

[52] Zalewski N.L., Tobin W.O. (2017). CLIPPERS. Curr. Neurol. Neurosci. Rep..

[53] Taieb G., Mulero P., Psimaras D., van Oosten B.W., Seebach J.D., Marignier R., Pico F., Rigau V., Ueno Y., Duflos C. (2019). CLIPPERS and its mimics: Evaluation of new criteria for the diagnosis of CLIPPERS. J. Neurol. Neurosurg. Psychiatry.

[54] Tobin W.O., Guo Y., Krecke K.N., Parisi J.E., Lucchinetti C.F., Pittock S.J., Mandrekar J., Dubey D., Debruyne J., Keegan B.M. (2017). Diagnostic criteria for chronic lymphocytic inflammation with pontine perivascular enhancement responsive to steroids (CLIPPERS). Brain.

[55] Taieb G., Allou T., Labauge P. (2017). Therapeutic Approaches in CLIPPERS. Curr. Treat. Options Neurol..

[56] Rempe T., Becktepe J.S., Metz I., Brück W., Stürner K.H., Deuschl G., Berg D., Baron R., Zeuner R., Leypoldt F. (2019). A case of CLIPPERS syndrome responsive to tocilizumab. Neurol. Neuroimmunol. Neuroinflamm..

[57] De Graaff H.J., Wattjes M.P., Rozemuller-Kwakkel A.J., Petzold A., Killestein J. (2013). Fatal B-cell lymphoma following chronic lymphocytic inflammation with pontine perivascular enhancement responsive to steroids. JAMA Neurol..

[58] Akman-Demir G., Serdaroglu P., Tasçi B. (1999). Clinical patterns of neurological involvement in Behçet’s disease: Evaluation of 200 patients. The Neuro-Behçet study group. Brain.

[59] Moragas M., Martínez-Yélamos S., Majós C., Fernández-Viladrich P., Rubio F., Arbizu T. (2011). Rhombencephalitis: A series of 97 patients. Medicine.

[60] Kidd D., Steuer A., Denman A.M., Rudge P. (1999). Neurological complications in Behçet’s syndrome. Brain.

[61] Fujimori J., Takahashi T., Matsumoto Y., Fujihara K., Takai Y., Misu T., Nakashima I. (2019). Two Japanese cases of anti-MOG antibody-associated encephalitis that mimicked neuro-Behçet’s disease. J. Neuroimmunol..

[62] Hajj-Ali R.A., Calabrese L.H. (2009). Central nervous system vasculitis. Curr. Opin. Rheumatol..

[63] Matsui Y., Takenouchi T., Narabayashi A., Ohara K., Nakahara T., Takahashi T. (2016). Childhood Sjögren syndrome presenting as acute brainstem encephalitis. Brain Dev..

[64] Chen J., Wang L., He L., Yi X., Yan Z. (2015). Teaching NeuroImages: Primary Sjögren syndrome presenting as isolated lesion of medulla oblongata. Neurology.

[65] Delalande S., de Seze J., Fauchais A.L., Hachulla E., Stojkovic T., Ferriby D., Dubucquoi S., Pruvo J.P., Vermersch P., Hatron P.Y. (2004). Neurologic manifestations in primary Sjögren syndrome: A study of 82 patients. Medicine.

[66] Kumar S., Sharma N., Sharma A., Mahi S., Bhalla A., Varma S. (2009). A case of systemic lupus erythematosus with extensive brain stem involvement. Clin. Rheumatol..

[67] Delèvaux I., André M., Marroun I., Lamaison D., Piette J.C., Aumaître O. (2005). Intractable hiccup as the initial presenting feature of systemic lupus erythematosus. Lupus.

[68] Shahmohammadi S., Doosti R., Shahmohammadi A., Mohammadianinejad S.E., Sahraian M.A., Azimi A.R., Harirchian M.H., Asgari N., Naser Moghadasi A. (2019). Autoimmune diseases associated with neuromyelitis optica spectrum disorders: A literature review. Mult. Scler. Relat. Disord..

[69] Zhong Y.H., Zhong Z.G., Zhou Z., Ma Z.Y., Qiu M.Y., Peng F.H., Zhang W.X. (2017). Comparisons of presentations and outcomes of neuromyelitis optica patients with and without Sjögren’s Syndrome. Neurol. Sci..

[70] Marino A., Narula S., Lerman M.A. (2017). First pediatric patient with neuromyelitis optica and Sjögren syndrome successfully treated with tocilizumab. Pediatr. Neurol..

[71] Dubey D., Wilson M.R., Clarkson B., Giannini C., Gandhi M., Cheville J., Lennon V.A., Eggers S., Devine M.F., Mandel-Brehm C. (2020). Expanded Clinical Phenotype, Oncological Associations, and Immunopathologic Insights of Paraneoplastic Kelch-like Protein-11 Encephalitis. JAMA Neurol..

[72] Dubey D., Kryzer T., Guo Y., Clarkson B., Cheville J.C., Costello B.A., Leibovich B.C., Algeciras-Schimnich A., Lucchinnetti C., Hammami M.B. (2021). Leucine Zipper 4 Autoantibody: A Novel Germ Cell Tumor and Paraneoplastic Biomarker. Ann Neurol..

[73] Mandel-Brehm C., Dubey D., Kryzer T.J., O′Donovan B.D., Tran B., Vazquez S.E., Sample H.A., Zorn K.C., Khan L.M., Bledsoe I.O. (2019). Kelch-like Protein 11 Antibodies in Seminoma-Associated Paraneoplastic Encephalitis. N. Engl. J. Med..

[74] Graus F., Vogrig A., Muñiz-Castrillo S., Antoine J.G., Desestret V., Dubey D., Giometto B., Irani S.R., Joubert B., Leypoldt F. (2021). Updated Diagnostic Criteria for Paraneoplastic Neurologic Syndromes. Neurol. Neuroimmunol Neuroinflamm..

[75] Dalmau J., Rosenfeld M.R. (2008). Paraneoplastic syndromes of the CNS. Lancet Neurol..

[76] Saiz A., Bruna J., Stourac P., Vigliani M.C., Giometto B., Grisold W., Honnorat J., Psimaras D., Voltz R., Graus F. (2009). Anti-Hu-associated brainstem encephalitis. J. Neurol. Neurosurg. Psychiatry.

[77] Lee K.S., Higgins M.J., Patel B.M., Larson J.S., Rady M.Y. (2006). Paraneoplastic coma and acquired central alveolar hypoventilation as a manifestation of brainstem encephalitis in a patient with ANNA-1 antibody and small-cell lung cancer. Neurocrit. Care.

[78] Dalmau J., Graus F., Villarejo A., Posner J.B., Blumenthal D., Thiessen B., Saiz A., Meneses P., Rosenfeld M.R. (2004). Clinical analysis of anti-Ma2-associated encephalitis. Brain.

[79] Pittock S.J., Lucchinetti C.F., Lennon V.A. (2003). Anti-neuronal nuclear autoantibody type 2: Paraneoplastic accompaniments. Ann. Neurol..

[80] Sutton I.J., Barnett M.H., Watson J.D., Ell J.J., Dalmau J. (2002). Paraneoplastic brainstem encephalitis and anti-Ri antibodies. J. Neurol..

[81] Lambeck J., Hieber M., Dreßing A., Niesen W.D. (2019). Central pontine myelinosis and osmotic demyelination syndrome. Dtsch. Ärzteblatt Int..

[82] Biousse V., Newman N.J., Chang G.Y. (2004). Brainstem involvement in hypertensive encephalopathy: Clinical and radiological findings. Neurology.

[83] Vasan S., Kumar A. (2020). Wernicke encephalopathy. Statpearls.

